# Preoperative anemia is associated with increased length of stay in adult spinal deformity surgery: evaluation of a large single-center patient cohort and future suggestions for patient optimization

**DOI:** 10.1007/s43390-024-01003-w

**Published:** 2024-11-07

**Authors:** Mert Marcel Dagli, Connor A. Wathen, Joshua L. Golubovsky, Yohannes Ghenbot, John D. Arena, Gabrielle Santangelo, Jonathan Heintz, Zarina S. Ali, William C. Welch, Jang W. Yoon, Vincent Arlet, Ali K. Ozturk

**Affiliations:** 1 Department of Neurosurgery, Perelman School of Medicine, University of Pennsylvania, 801 Spruce Street, Philadelphia, PA 19107 USA; 2 Biostatistics Analysis Center, Center for Clinical Epidemiology and Biostatistics, Perelman School of Medicine, University of Pennsylvania, Philadelphia, PA USA; 3 Department of Orthopaedic Surgery, Perelman School of Medicine, University of Pennsylvania, Philadelphia, PA USA

**Keywords:** Blood loss, Surgical, Retrospective studies, Spinal curvatures, Length of stay, Prognosis, Anemia, Spinal fusion, Adult, Humans, Linear models, Hemoglobin, Intensive care units, Spine

## Abstract

**Purpose:**

This study aimed to investigate the relationship of preoperative hemoglobin levels as an independent prognostic factor for hospital and intensive care unit (ICU) length of stay (LOS) in patients undergoing surgery for adult spinal deformity (ASD), with the intent of determining whether there exists a correlation and enhancing patient preoperative optimization protocols.

**Methods:**

The authors reviewed consecutive patients who underwent elective thoracolumbosacral posterior spinal fusion (PSF) involving six or more vertebrae for ASD from January 1, 2013, to December 13, 2021, with a minimum follow-up period of two years. This study primarily investigated the association of preoperative hemoglobin levels with hospital and ICU LOS. To analyze the data, both unadjusted and adjusted generalized linear models (GLM), incorporating cubic splines for non-linear variables, were applied.

**Results:**

A total of 598 patients were included. GLMs for hospital and ICU LOS demonstrated nonlinear relationships with preoperative hemoglobin levels. Specifically, hospital LOS decreased with increasing preoperative hemoglobin until a significance threshold of 13.5 g/dl. Similarly, ICU LOS significantly decreased with increasing preoperative hemoglobin until 13.0 g/dl. Lower preoperative hemoglobin was associated with more perioperative transfusions, less likely discharge to home, and greater risk of reoperation.

**Conclusions:**

Preoperative anemia is an independent non-linear risk factor that significantly affects LOS, disposition, and outcomes after surgery for ASD. These findings advocate for a systemic preoperative approach and highlight the need for future research to improve postoperative outcomes and reduce hospital resource utilization.

**Level of evidence:**

IV.

## Introduction

Adult spinal deformity (ASD) has become increasingly prevalent as the US population ages, with a prevalence of up to 70% in the elderly [1]. Corresponding with this increase, the volume of surgical interventions for deformity correction has continued to rise [2]. Alongside the rise in prevalence of ASD and advances in surgical techniques, there has been a growing focus on improvement of postoperative outcomes in these complex patients via preoperative optimization, particularly in the elderly [3]. Preoperative anemia has been identified as a potentially modifiable risk factor associated with worsened postoperative outcomes [4–7].

Significant blood loss is often an inevitable complication of spinal deformity surgery and is associated with increased rates of perioperative transfusion as well as extended length of stay (LOS) [8, 9]. There is a strong incentive to avoid transfusions, as perioperative transfusions in spine surgery have been associated with increased LOS, reoperation, infection, and other postoperative complications [10]. Therefore, techniques to reduce intraoperative blood loss (such as administration of tranexamic acid (TXA) [11] and use of radiofrequency bipolar sealer systems [12]) and optimization of patients’ preoperative hemoglobin have received increased attention. Anemia has been identified as a preoperative risk factor for delirium in elderly patients undergoing fusion, an outcome that significantly impacts hospital LOS and associated costs [13]. Preoperative hemoglobin levels have also been found to inversely correlate with hospital LOS after spinal fusions and risk of major complications and infection in patients with hemoglobin values correlating with severe anemia [14, 15].

In this study, we evaluated a large cohort of patients who underwent thoracolumbar posterior spinal fusion (PSF) for ASD. We hypothesized that patients with lower preoperative hemoglobin levels would experience increased LOS, which was our primary outcome, even after adjusting for other comorbidities, age, and levels of fusion. Secondary, we explored the relationship between preoperative hemoglobin levels and perioperative outcomes. We leveraged our findings to propose a new protocol to improve our existing optimization procedures by incorporating preoperative hemoglobin, which we aim to investigate in future studies.

## Methods

### Guidelines

The design and reporting of this study were supported by the Strengthening the Reporting of Observational Studies in Epidemiology (STROBE) and the Transparent Reporting of a Multivariable Prediction Models for Individual Prognosis or Diagnosis (TRIPOD) guidelines [16, 17].

### Data source

The review of Electronic Health Records (EHR) was approved by the Institutional Review Board (IRB 848854). This study was limited to a retrospective review, and individual patient consent was waived. We developed a comprehensive institutional spinal deformity database using a multifaceted data collection strategy that combined automated variable extraction via reports from our institutional Data Analytics Center (DAC) and a thorough manual review of EHRs.

### Patient selection

Database entries with surgery dates between January 1, 2013, and December 13, 2021, were reviewed to identify adult patients (age ≥ 18 years) with a diagnosis of ASD undergoing elective thoracolumbosacral PSF of six or more vertebrae and a minimum follow-up of two years. The exclusion criteria were age < 18 years, PSF of less than six levels, staged procedures, less than two-year follow-up, surgeries with a cervical component, and non-deformity surgical indication, such as tumor, trauma, or infection.

### Outcomes

The primary objective of this study was to evaluate the association of preoperative hemoglobin levels with hospital and intensive care unit (ICU) LOS. Secondary outcomes included the exploration of the relationships between preoperative hemoglobin levels and perioperative outcomes.

### Variables

The following variables were extracted for each eligible patient: age (years), sex, race, smoking status, preoperative hemoglobin [grams per deciliter {g/dL}], body mass index (BMI) [kilograms per square meter (kg/m^2^}], Charleson Comorbidy Index (CCI) score, operative time (minutes), number of vertebrae fused with PSF, posterior column osteotomy (PCO) use, number of vertebrae PCO performed on, 3-column osteotomy (3CO) use, number of vertebrae 3CO performed on, estimated blood loss (EBL) [milliliter {mL}], intraoperative complications (dural, vascular, and nerve injury), intraoperative transfusion requirements (packed red blood cells (pRBC), cell saver, plasma, platelets) [milliliter {mL}], TXA use [milliliter {mL}], postoperative complications (cardiac, renal, pulmonary, neurologic, surgical site, vascular, other), postoperative transfusion requirements (pRBC, plasma, platelets) [milliliter {mL}], hospital LOS (days), ICU LOS (days), discharge disposition (home, acute rehabilitation, skilled nursing facility (SNF)), readmission (30 days, 60 days, 90 days, 1 year, 2 years), and reoperation ( 30 days, 60 days, 90 days, 1 year, 2 years). Covariate selection was judged relevant by prior work and a panel of clinical experts, including three neurosurgeons, a research fellow, and a statistician for adjusted analysis, including age, sex, race, BMI, smoking status, ASA score, CCI score, operative time, number of vertebrae fused with PSF, number of vertebrae 3CO performed on, number of vertebrae PCO performed on, intraoperative pRBC transfusion, intraoperative cell saver return, intraoperative dural complication, and postoperative complication. Factors determining timing of discharge were standard between patients and included obtaining requisite postoperative imaging, meeting criteria for drain removal, not requiring intravenous pain medications, and completing inpatient courses of physical and occupational therapy as recommended by the therapy teams until they were deemed fit for discharge to either home, acute rehabilitation, or skilled nursing facilities. The intraoperative and postoperative transfusion protocols for fresh frozen plasma included administration for an INR > 1.4 or elevated PTT, especially in the presence of ongoing hemoglobin decline or hypotension. Platelet transfusion was indicated when platelet levels drop below 100,000 platelets/µL.

### Missing data

The overall incidence of incomplete data was less than 5%, with 27 records (4.52%) missing at least one variable. We utilized a complete-case analysis approach.

### Statistics

The baseline characteristics of the cohort were summarized using descriptive statistics. Unadjusted and adjusted models were used to elucidate the relationships between preoperative hemoglobin levels and healthcare utilization (LOS and ICU LOS). Smoking status (binary: non-smoker, current, or former smoker) and CCI score (binary: 0–1, 2–4) were dichotomized to streamline the analysis and enhance interpretability. For both LOS and ICU LOS models, multiple residual distributions were assessed. Based on −2 log-likelihood tests and visual inspection of histograms and residuals, a gamma distribution fit best in all cases. For LOS, generalized linear models (GLM) with a gamma residual distribution and log-link function were utilized. For ICU LOS, we used a two-part modeling procedure. The first part modeled a logistic regression with a binary outcome: ICU stay versus no ICU stay, while the second part modeled a GLM with a gamma residual distribution and log-link function. Numerical covariates were tested for non-linearity, and cubic splines with three knots were included for significantly non-linear covariates. Notably, preoperative hemoglobin level, our covariate of interest, was significantly nonlinear for both LOS and ICU LOS. Statistical analysis was performed using Stata v18.0 (StataCorp LLC) and figures were generated with Python 3.7 (Python Foundation).

## Results

### Patient demographics and baseline characteristics

**Fig. 1:**
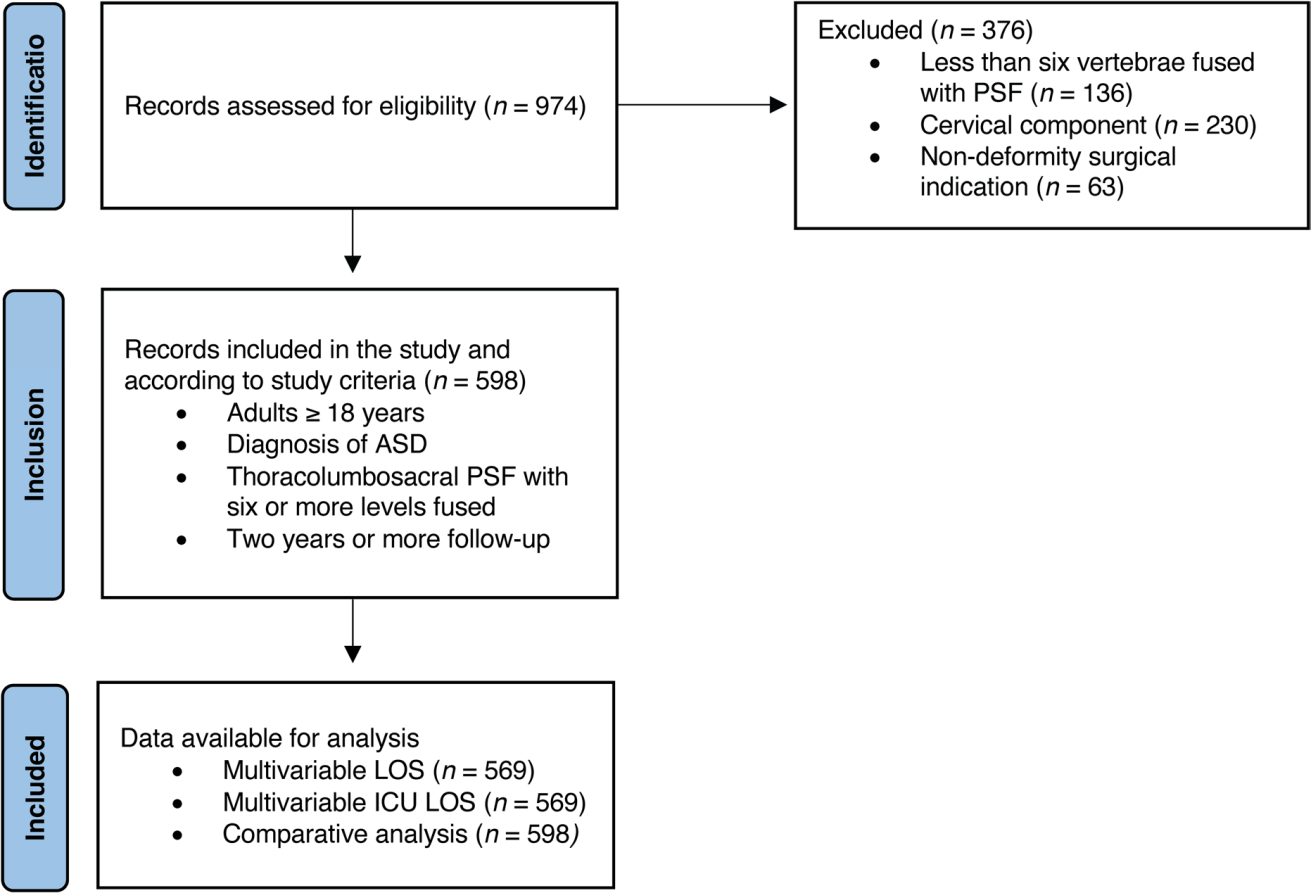
Flow chart of study

**Fig. 2:**
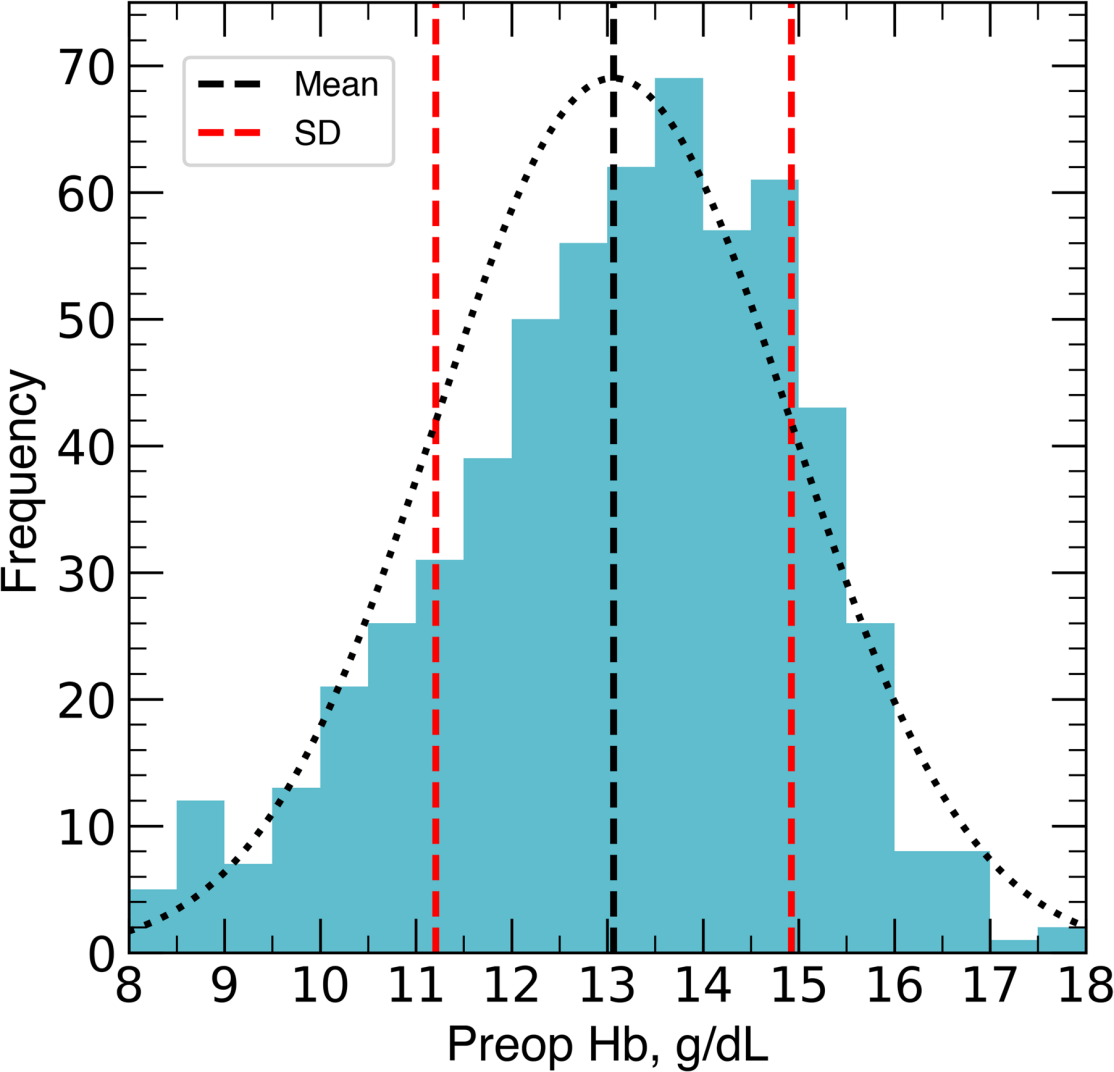
Histogram of preoperative hemoglobin illustrating normal distribution, mean, and standard deviation (SD). The black and red dashed lines indicate mean and SD, respectively

A total of 598 patients were included in this study (Fig. 1). Mean age was 59.9 ± 14.9 years and 389 (65.1%) were female (Table 1). The mean preoperative hemoglobin was 13.1 ± 1.9 g/dL (Fig. 2).

**Table 1:** Baseline characteristics of study population, *n* = 598

Variable	*n* (%) or Mean ± SD
Age, years	59.9 ± 14.9
Sex	
Female	389 (65.1)
Male	209 (34.9)
Race	
White	508 (85)
Black	51 (8.5)
Hispanic	15 (2.5)
Other	24 (4)
Smoking status	
Smoker	56 (9.4)
Ex-smoker	217 (36.3)
Non-smoker	318 (53.2)
Preoperative hemoglobin, g/dL	13.1 ± 1.9
BMI	28 ± 6.3
ASA	
ASA 0	1 (0.2)
ASA 1	14 (2.4)
ASA 2	310 (51.9)
ASA 3	267 (44.7)
ASA 4	6 (1)
CCI	
CCI 0	88 (14.7)
CCI 1	70 (11.7)
CCI 2	186 (31.1)
CCI 3	186 (31.1)
CCI 4	55 (9.2)

Data are mean ± standard deviation or number of patients (%)

*ASA* American Society of Anesthesiologists, *BMI* body-mass-index

### Perioperative data and outcomes

**Fig. 3:**
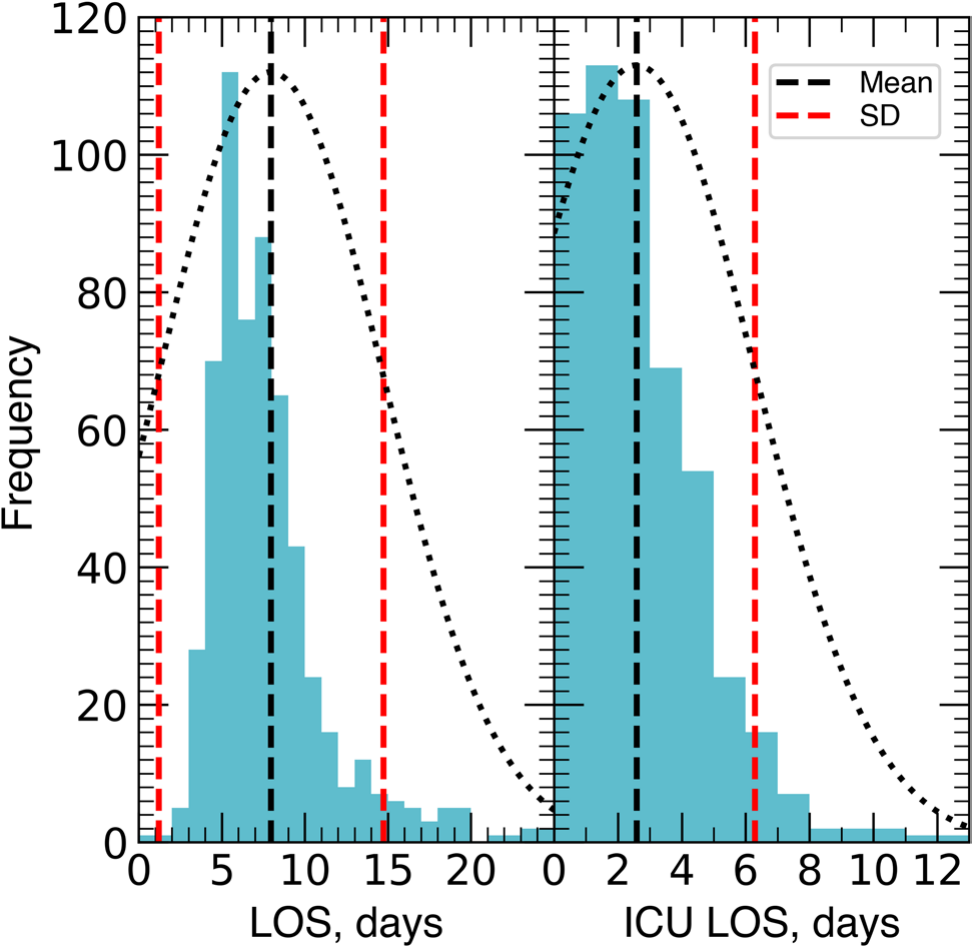
Combination histogram of hospital length of stay (LOS, left) and intensive care unit length of stay (ICU LOS, right) illustrating non-normal distribution, mean, and standard deviation (SD). The black and red dashed lines indicate mean and SD, respectively

The mean number of vertebrae fused was 10.7 ± 3. 59 patients had 3COs at a mean of 1.3 ± 0.5 levels, and 379 patients underwent PCOs at a mean of 4.1 ± 2.1 levels (Table 2). The most common intraoperative complication was dural tear (n = 42, 7%), followed by vascular injury (n = 4, 0.7%). Additionally, one patient had complete loss of evoked motor potentials on the right side without postoperative sequelae after resolution. Of the 257 patients (43%) who were infused with TXA, 90 (15.1%) received bolus-only, 25 (4.2%) received infusion with maintenance dose-only, and 142 (23.7%) received a loading dose followed by continuous infusion. 134 patients (22.4%) received anterior lumbar interbody fusion (ALIF) with a mean of 0.6 ± 1.1 levels fused, 32 patients (5.4%) received transforaminal lumbar interbody fusion (TLIF) with a mean of 0.1 ± 0.5 levels fused, and 22 patients received lateral lumbar interbody fusion (LLIF) with a mean of 0.1 ± 0.5 levels fused. Average LOS in our cohort was 8 ± 6.8 days, with average ICU LOS of 2.6 ± 3.7 days (Table 3, Fig. 3).

**Table 2:** Intraoperative details, *n* = 598

Variable	*n* (%) or Mean ± SD
Posterior spinal fusion, levels	10.7 ± 3
3-column osteotomy	59 (9.9)
Levels	1.3 ± 0.5
Posterior column osteotomy	379 (63.4)
Levels	4.1 ± 2.1
Operative time, minutes	509.8 ± 126.1
Tranexamic acid use	257 (43.0)
Estimated blood loss, mL	1334.8 ± 962.3
≥ 1 intraoperative complication	51 (8.5)
Dural	42 (7)
Vascular	4 (0.7)
Nerve injury	1 (0.2)
Intraoperative transfusion	520 (87)
pRBC, mL	698.6 ± 768.7
Cell saver return, mL	271.5 ± 296.2
Plasma, mL	166.3 ± 411.2
Platelets, mL	47.9 ± 132.6

Data are mean ± standard deviation or number of patients (%)

*pRBC* packed red blood cells

**Table 3:** Postoperative complications and outcomes, *n* = 598

Variable	*n* (%) or Mean ± SD
≥ 1 postoperative complication	355 (59.4)
Cardiac	217 (36.3)
Renal	76 (12.7)
Pulmonary	102 (17.1)
Neurological	76 (12.7)
Surgical site	7 (1.2)
Vascular	51 (8.5)
Other	117 (19.6)
Postoperative transfusion	234 (39.1)
pRBC, mL	226.5 ± 380.6
Plasma, mL	12.1 ± 112.1
Platelets, mL	12.7 ± 110.6
LOS, days	8 ± 6.8
ICU LOS, days	2.6 ± 3.7
Disposition	
Home	240 (40.1)
Acute rehabilitation	265 (44.3)
Skilled nursing facility	84 (14.1)
Unplanned readmission	
Within 30-days	84 (14.1)
Within 90-days	93 (15.6)
Within 1 year	121 (20.2)
Within 2 years	236 (39.5)
≥ 1 reoperation	81 (13.6)
Within 30 days	27 (4.5)
Within 60 days	36 (6)
Within 90 days	39 (6.5)
Within 1 year	63 (10.5)
Within 2 years	77 (12.9)

Data are mean ± standard deviation or number of patients (%)

*ICU* intensive care unit, *LOS* length of stay, *pRBC* packed red blood cells

### Healthcare utilization

**Fig. 4:**
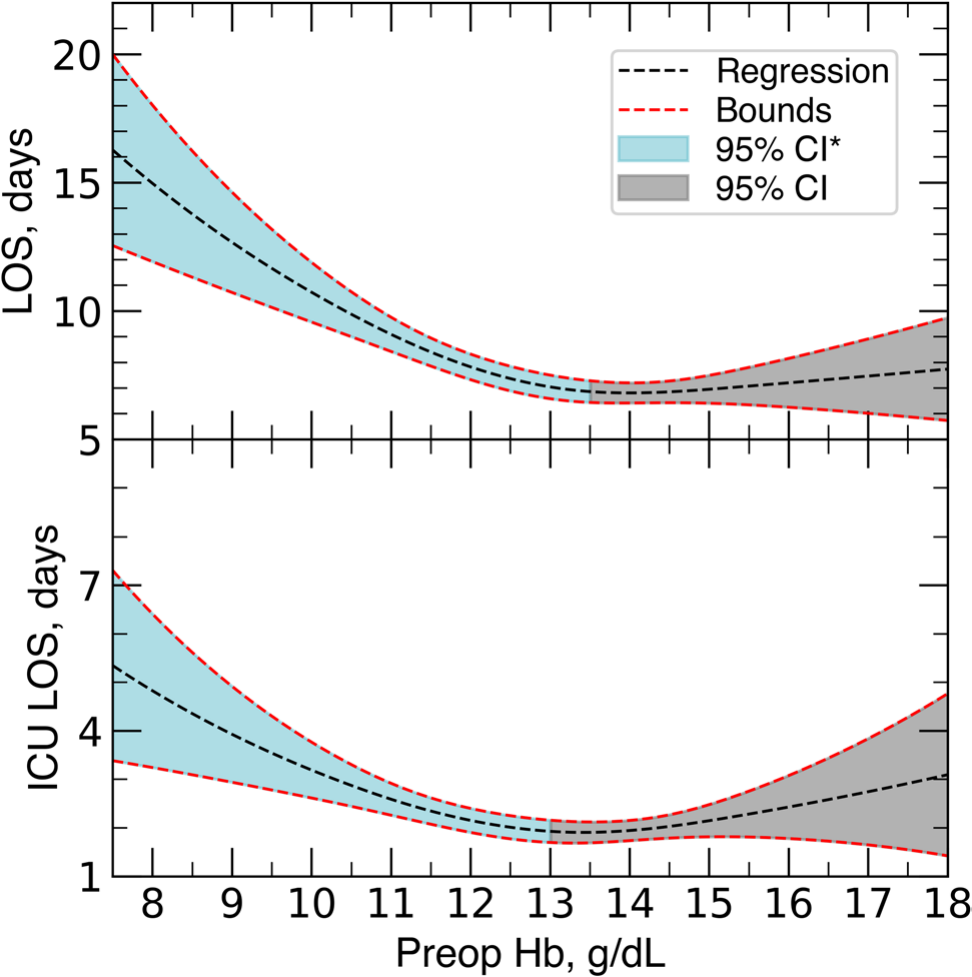
Regression models for length of stay (LOS, top) and intensive care unit length of stay (ICU LOS, bottom) using preoperative hemoglobin as predictor. The black and red dashed lines indicate the regression slope (regression) and bounds (upper and lower bounds of the confidence interval), respectively. The blue area indicates the significant values within the 95% confidence interval (95% CI^*^) before the significance threshold, while they grey area indicates the non-significant area within 95% CI (95% CI)

The unadjusted regression model for LOS showed a significant association between preoperative hemoglobin levels and LOS for values of preoperative hemoglobin less than 13.6 g/dL. The adjusted regression model for LOS showed a significant association between preoperative hemoglobin and LOS for preoperative hemoglobin less than 13.5 g/dL (Table 4 and Fig. 4). Specifically, for all preoperative hemoglobin values less than 13.5 g/dL, we expect LOS to decrease as preoperative hemoglobin increases; however, the expected change in LOS approaches 0 as preoperative hemoglobin increases. After 13.5 g/dL, we see a relatively constant expected length of stay. A similar model was created for ICU LOS (Table 5), with the adjusted regression model showing a significant association between preoperative hemoglobin and ICU LOS for preoperative hemoglobin values less than 13.0 g/dL (Fig. 4). The numeric values of the expected hospital and ICU LOS based on preoperative hemoglobin unit increases of 1.0 g/dL are in Table 6.

**Table 4:** Adjusted regression model parameters for hospital length of stay, *n* = 569

Variable	$\beta$ coefficient	95% CI	SE	*P* value
Preoperative hemoglobin spline 1, g/dL	−0.167	−0.220 to –0.113	0.027	< 0.001^*^
Preoperative hemoglobin spline 2, g/dL	0.116	0.055–0.176	0.031	< 0.001^*^
Age, years	0.001	−0.004–0.007	0.003	0.624
BMI spline 1, kg/m^2^	−0.019	−0.038–< 0.001	0.010	0.056
BMI spline 2, kg/m^2^	0.027	0.004–0.050	0.012	0.023^*^
High CCI	−0.025	−0.199–0.149	0.089	0.778
Female	−0.062	−0.165–0.042	0.053	0.246
Posterior spinal fusion, levels	0.019	0.001–0.036	0.009	0.034^*^
Operative time, minutes	0.001	0.001–0.002	< 0.001	< 0.001^*^
Current or former smoker	−0.055	−0.149–0.039	0.048	0.250
3-column osteotomy 1 level	−0.029	−0.216–0.158	0.096	0.763
3-column osteotomy 2 level	0.109	−0.183–0.401	0.149	0.465
Posterior column osteotomy, levels	−0.011	−0.032–0.010	0.011	0.307
Intraoperative transfusion	0.114	0.007–0.222	0.055	0.037^*^
Postoperative transfusion	−0.024	−0.119–0.072	0.049	0.624
Black	0.015	−0.151–0.181	0.085	0.860
Hispanic	0.296	−0.001–0.594	0.152	0.051
Other Race	−0.058	−0.303–0.188	0.125	0.646
ASA 3 + score	0.286	0.189–0.384	0.050	< 0.001^*^
Postoperative complication	0.040	−0.146–0.225	0.095	0.675
Intraoperative dural complication	−0.109	−0.214 to –0.003	0.054	0.043^*^
Intraoperative cell saver return	3.784	3.110–4.458	0.344	< 0.001^*^
Intercept	0.286	0.189–0.384	0.050	< 0.001^*^

Intercept is the point where the regression line meets the y-axis

Splines represent smooth, flexible curves used in nonlinear models

*BMI* body-mass-index, *CCI* Charlson-comorbidity-index, *CI* confidence interval, *SE* standard error

^*^P < 0.05

**Table 5:** Adjusted regression model parameters for intensive care unit length of stay, *n* = 569

Variable	Part 1: logistic regression					Part 2: generalized linear model (Gamma distribution, log link)			
	$\beta$ coefficient	95% CI		SE	*P* Value	$\beta$ coefficient	95% CI	SE	*P* value
Preoperative hemoglobin spline 1, g/dL	−0.162	−0.436–0.113		0.140	0.248	−0.188	−0.270 to –0.106	0.042	< 0.001^*^
Preoperative hemoglobin spline 2, g/dL	0.029	−0.264–0.323		0.150	0.845	0.191	0.091–0.291	0.051	< 0.001^*^
Age, years	−0.021	−0.046–0.005		0.013	0.110	0.007	−0.002–0.016	0.005	0.135
BMI spline 1, kg/m^2^	0.004	−0.033–0.041		0.019	0.832	0.004	−0.009–0.016	0.006	0.564
High CCI	0.636	−0.167–1.438		0.409	0.120	−0.046	−0.339–0.247	0.149	0.756
Female	−0.431	−0.941–0.078		0.260	0.097	−0.080	−0.245–0.085	0.084	0.344
Posterior spinal fusion, levels	0.070	−0.017–0.157		0.044	0.115	0.012	−0.015–0.039	0.014	0.380
Operative time, minutes	0.004	0.002–0.006		0.001	< 0.001	< 0.001	< 0.001–0.001	< 0.001	0.289
Current or former smoker	0.146	−0.297–0.589		0.226	0.518	−0.183	−0.337 to –0.028	0.079	0.021^*^
3-column osteotomy 1 level	−0.405	−1.365–0.554		0.490	0.408	0.041	−0.260–0.342	0.153	0.791
3-column osteotomy 2 level	−0.094	−1.776–1.589		0.859	0.913	0.059	−0.390–0.507	0.229	0.798
Posterior column osteotomy, levels	−0.052	−0.153–0.048		0.051	0.306	−0.027	−0.062–0.008	0.018	0.129
Intraoperative pRBC transfusion	0.723	0.240–1.206		0.246	0.003	0.257	0.074–0.439	0.093	0.006^*^
Postoperative transfusion	0.041	−0.430–0.512		0.240	0.864	−0.048	−0.204–0.107	0.079	0.543
Black	−0.328	−1.080–0.424		0.384	0.393	0.167	−0.109–0.443	0.141	0.235
Hispanic	1.228	−0.883–3.339		1.077	0.254	−0.178	−0.612–0.257	0.222	0.423
Other Race	−1.041	−2.096–0.014		0.538	0.053	−0.238	−0.674–0.199	0.223	0.286
ASA 3 + score	0.073	−0.417–0.563		0.250	0.771	0.112	−0.047–0.272	0.081	0.167
Postoperative complication	1.092	0.646–1.538		0.228	< 0.001	0.262	0.096–0.427	0.084	0.002^*^
Intraoperative dural complication	0.347	−0.686–1.380		0.527	0.510	0.062	−0.229–0.353	0.149	0.677
Intraoperative cell saver return	0.153	−0.335–0.642		0.249	0.538	−0.183	−0.360 to –0.006	0.090	0.042^*^
Intercept	1.979	−1.442–5.400		1.745	0.257	3.098	2.046–4.149	0.536	< 0.001^*^

Intercept is the point where the regression line meets the y-axis

Splines represent smooth, flexible curves used in nonlinear models

*BMI* body-mass-index, *CCI* Charlson-comorbidity-index, *CI* confidence interval, *pRBC* packed red blood cells, *SE* standard error

^*^P < 0.05

**Table 6:** Change in expected hospital and intensive care unit length of stays based on preoperative hemoglobin levels, *n* = 569

PreopHb	LOS	SE	95% CI	ICU LOS	SE	95% CI
7.5	16.3	1.9	12.7–20.5	5.3	1.0	3.3–7.3
8.5	13.8	1.3	11.5–16.6	4.4	0.6	3.1–5.6
9.5	11.7	0.8	10.3–13.4	3.5	0.4	2.8–4.3
10.5	9.9	0.4	9.1–10.9	2.9	0.2	2.4–3.3
11.5	8.4	0.3	7.9–9.0	2.3	0.1	2.1–2.6
12.5	7.4	0.2	6.9–7.9	2.0	0.1	1.8–2.3
13.5	6.9	0.2	6.4–7.3	1.9	0.1	1.7–2.1
14.5	6.8	0.2	6.4–7.2	2.0	0.1	1.8–2.3
15.5	7.1	0.4	6.3–7.7	2.3	0.2	1.8–2.7
16.5	7.3	0.6	6.0–8.4	2.6	0.4	1.7–3.3
17.5	7.6	0.9	5.7–9.2	2.9	0.7	1.5–4.1

*CI* confidence interval, *ICU* intensive care unit, *LOS* length of stay, *PreopHb* preoperative hemoglobin, *SE* standard error

### Relationship of preoperative hemoglobin with perioperative outcomes

Based on stratification using the lower significance threshold of 13.0 g/dL of the ICU LOS model, there were significant increases in intraoperative and perioperative pRBC, plasma, and platelet transfusion volumes in the lower hemoglobin groups (Table 7). Additionally, there were significantly higher rates of discharge to home and lower rates of reoperation within 1 year and 2 years in the higher preoperative hemoglobin group.

**Table 7:** Comparison of perioperative outcomes based on preoperative hemoglobin level thresholds, *n* = 598

Variables	Preop Hb > 13.0	Preop Hb ≤ 13.0	*P* value
n	323	275	
Operative time, minutes	506.1 ± 124.4	514.1 ± 128.1	0.446
Tranexamic acid use	149 (46.1)	108 (39.3)	0.108
Estimated blood loss, mL	1297.3 ± 928.9	1378.7 ± 1000.1	0.306
≥ 1 intraoperative complication	32 (9.9)	19 (6.9)	0.246
Dural	25 (7.7)	17 (6.2)	0.560
Vascular	3 (0.9)	1 (0.4)	0.629
Nerve injury	1 (0.3)	0 (0.0)	1.000
Intraoperative transfusion volumes	268 (83.0)	252 (91.6)	0.003^*^
pRBC, mL	525.6 ± 702.5	901.7 ± 794.2	< 0.001^*^
Cell saver return, mL	283.6 ± 305.2	257.3 ± 285.2	0.277
Plasma, mL	123.5 ± 389.6	216.4 ± 430.5	0.006^*^
Platelets, mL	27.1 ± 95.6	72.3 ± 162.7	< 0.001^*^
≥ 1 postoperative complication	189 (58.5)	165 (60.0)	0.776
Cardiac	129 (39.9)	88 (32.0)	0.054
Renal	46 (14.2)	30 (10.9)	0.273
Pulmonary	53 (16.4)	49 (17.8)	0.728
Neurological	39 (12.1)	37 (13.5)	0.703
Surgical site	4 (1.2)	3 (1.1)	1.000
Vascular	30 (9.3)	21 (7.6)	0.566
Other	66 (20.4)	51 (18.5)	0.634
Postoperative transfusion volumes	121 (37.5)	113 (41.1)	0.411
pRBC, mL	218.9 ± 360.2	235.5 ± 403.7	0.599
Plasma, mL	15.8 ± 139.3	7.8 ± 67.4	0.363
Platelets, mL	16.1 ± 122.0	8.7 ± 95.5	0.408
Perioperative transfusion volumes			
pRBC, mL	744.5 ± 826.9	1137.1 ± 913.9	< 0.001^*^
Plasma, mL	139.3 ± 427.1	224.3 ± 448.1	0.019^*^
Platelets, mL	43.2 ± 170.9	81.0 ± 208.7	0.017^*^
LOS, days	6.7 ± 4.4	9.4 ± 8.5	< 0.001
ICU LOS, days	2.2 ± 3.1	3.1 ± 4.3	0.007
Disposition			
Home	145 (44.9)	95 (34.5)	0.013^*^
Acute rehabilitation	142 (44.0)	123 (44.7)	0.916
Skilled nursing facility	35 (10.8)	49 (17.8)	0.020
Other	1 (0.3)	8 (2.9)	0.014
30-day readmission	46 (14.2)	38 (13.8)	0.976
≥ 1 reoperation	35 (10.8)	46 (16.7)	0.048
30-day reoperation	11 (3.4)	16 (5.8)	0.223
60-day reoperation	15 (4.6)	21 (7.6)	0.174
90-day reoperation	17 (5.3)	22 (8.0)	0.236
1-year reoperation	26 (8.0)	37 (13.5)	0.044^*^
2-year reoperation	33 (10.2)	44 (16.0)	0.047^*^

*ICU* intensive care unit, *LOS* length of stay, *pRBC* packed red blood cells

^*^P < 0.05

## Discussion

ASD correction is associated with high postoperative morbidity and intraoperative blood loss, thus increasing the desire to establish clear protocols for perioperative optimization [3]. Preoperative modifiable factors are of particular interest. Numerous factors have been identified in the spine surgery literature, including osteoporosis, functional status, obesity, nutrition, smoking, Hemoglobin A1c, preoperative hemoglobin, and preoperative opioid use [18]. Unfortunately, the disability that accompanies ASD often limits adequate optimization of factors such as functional status, obesity, and preoperative opioid use. Novel medications that assist in weight loss may improve the current deficiencies. Smoking and diabetes have long been recognized as risk factors for poor outcomes across all fields of surgery, and their role in preoperative optimization is well established. With respect to osteoporosis, investigations are ongoing for the use of medications such as teriparatide and bisphosphonates to improve bone mineral density preoperatively [19]. Conversely, although preoperative hemoglobin has been more extensively explored as a modifiable risk factor in orthopedic [4, 6], general [5, 20], cardiac [21, 22], and gynecological surgery [23], studies of its importance in ASD are limited [14, 15]. We hypothesized that low preoperative hemoglobin would be an independently modifiable risk factor that would lead to increased length of stay and worsened postoperative outcomes in patients undergoing thoracolumbar deformity correction surgery of six or more levels.

In our study, lower preoperative hemoglobin levels were significantly associated with increased perioperative transfusion volumes. Our findings demonstrate that preoperative hemoglobin levels below 13.0 are associated with increased overall and intensive care unit (ICU) length of stay (LOS), with a continued upward trend in LOS as preoperative hemoglobin values decrease below this threshold. Additionally, preoperative hemoglobin levels below 13.0 were associated with lower likelihood of discharge to home and greater likelihood of discharge to SNF, as well as greater rates of reoperation within 1 and 2 years. Meanwhile, preoperative hemoglobin levels below 13.5 were significantly associated with longer overall LOS.

Other variables noted to be associated with increased LOS in our cohort included BMI, number of levels fused, operative time, need for intraoperative transfusions, ASA score, and smoking (for ICU LOS only). Levels fused, operative time, and intraoperative transfusion volume are all correlated to complexity of surgery. These factors have been well established in literature as being associated with length of stay in spine surgery. Similarly, obesity has been tied to increased surgical times due to more extensive tissue exposure and worsened postoperative outcomes after spine surgery with higher rates of postoperative complications [24]. ASA is an overall surrogate for patient health and has been well described across surgical literature as a predictor of hospital LOS [25]. Lastly, smoking has been linked to increased rates of respiratory complications after surgery, thereby potentially necessitating longer ICU lengths of stay for prolonged intubation or need for more frequent respiratory therapies [26].

Low hemoglobin has been found to have a strong link to frailty, particularly in the elderly [27]. Low hemoglobin is tied to the concept of “inflammaging” [28], whereby aging is accompanied by an upregulation of the inflammatory immune response, a consequence of which is anemia secondary to free radical exposure. Younger anemic patients have the ability to compensate for decreased oxygen-carrying capacity secondary to decreased hemoglobin, but as patients age, their functional organ reserves also decline, reducing their overall functional status and increasing frailty [27]. Throughout the past decade, multiple frailty scores have been developed, of which anemia consistently features as a possibly modifiable factor [29, 30]. Anemia has also been linked to sarcopenia and poor physical performance, both factors which prolong recovery from surgery and lead to increased use of hospital resources [31, 32].

Anemia has had an evolving definition within population-based studies over the years. The commonly referenced values for anemia were established by the World Health Organization (WHO) in 1968, wherein in a cohort of patients < 65 years of age, anemia was defined as Hgb < 130 g/L for men and < 120 g/L for women [33]. Higher Hgb thresholds have been proposed by analyses of the National Health and Nutrition Examination Survey as well as the Scripps-Kaiser database [34, 35]. Further studies have evaluated the intersection of Hgb levels and outcomes to define anemia. These include thresholds of > 137 g/L for men and > 126 g/L for women in relation to improved survival in the Cardiovascular Health Study [36] and thresholds of > 130–150 g/L in women and > 140–170 g/L in men to avoid hospitalization and mortality [37]. Despite these investigations into thresholds for anemia, the standards defined by the WHO in 1968 remain the most prevalently referenced in modern literature and are supported by various experts, particularly in the elderly [38]. Most recently, a multi-national initiative known as Patient Blood Management (PBM) has been endorsed by the World Health Assembly and WHO, with the aim of detecting and optimally treating perioperative anemia. PBM expert panels have suggested a desirable Hgb cutoff of > 13 g/dL in both men and women in surgical populations [39]. This anemia threshold correlates closely with the findings in this study, wherein Hgb values < 13 and < 13.5 were associated with worse outcomes and longer ICU LOS and overall LOS respectively.

Anemia may be multifactorial, including those related to functional deficiencies, such as iron, folate, or cobalamin, or those secondary to other conditions, such as renal disease and chronic inflammation. Therefore, proper optimization of anemia in patients undergoing surgery should be comprehensively investigated by established anemia clinics, which have been demonstrated to improve postoperative outcomes [23]. A meta-analysis of administration of erythropoietin plus iron perioperatively in patients undergoing non-cardiac surgery (predominantly orthopedic, gastrointestinal, and gynecological surgeries) demonstrated that high-dose erythropoietin and iron reduced the need for perioperative transfusions and increased preoperative hemoglobin, but did not necessarily reduce LOS or postoperative complications [20]. Totonchi et al. similarly showed a reduction in the need for perioperative transfusions secondary to preoperative optimization with recombinant erythropoietin [22]. In a randomized controlled trial evaluating patients undergoing elective cardiac surgery in which patients were given either a placebo or combined treatment with iron, erythropoietin, B12, and folic acid on the day before surgery, Spahn et al. demonstrated that only an ultra-short treatment regimen was needed to reduce perioperative transfusions [21].

### Limitations

Although this study provides valuable insights into the association between preoperative anemia and increased LOS and ICU LOS in ASD surgery, it is imperative to acknowledge certain limitations. Although our study benefited from a large single-center cohort, it inherently limits the generalizability of our findings. The retrospective nature of the study, reliant on electronic health records (EHR), might introduce biases related to data completeness and accuracy. Importantly, our findings do not prove that preoperative correction of anemia will lead to shorter lengths of stay, or that preoperative hemoglobin levels are simply a surrogate of frailty or overall health. This needs to be studied carefully and prospectively. Despite these limitations, our study makes a significant contribution to the existing literature and is one of the few studies to rigorously investigate the impact of preoperative hemoglobin levels on the length of stay in ASD surgery. Further research and validation are necessary to arrive at definitive conclusions.

## Proposal for preoperative hemoglobin optimization in adult spinal deformity

We plan to institutionalize a preoperative optimization protocol for all patients undergoing ASD surgery in a similar fashion to the model established by Guinn et al. at Duke in orthopedic and gynecologic surgery populations [23]. All patients being considered for spinal deformity surgery at our institution were evaluated at our Spine Center, all generally > 60 days prior to scheduling of surgery if they were deemed to be appropriate surgical candidates. If patients are determined to be surgical candidates, they will have a complete blood count sent at the time of the initial Spine Center office visit to screen for anemia, and all patients will be referred to a nutrition clinic for optimization. In keeping with the results of our study, we will utilize a hemoglobin cutoff of 13 g/dL, which is a rounded threshold based on our results and correlating well with proposed cutoffs by the PBM. If patients fall below this cutoff, they will be referred to our institution’s hematology/internal medicine clinics for a comprehensive anemia workup, and a full panel of labs to further evaluate the underlying etiology of anemia will be ordered. These include ferritin, iron levels, total iron-binding capacity, folate, vitamin B12, reticulocyte count, and creatinine. Based on the underlying cause of anemia, patients will be treated accordingly in a 30–60 day protocol with a combination of oral and intravenous regimens as needed (Guinn et al. showed limited benefit to preoperative hemoglobin levels or outcomes after 60 days of therapy). Hemoglobin levels will be repeated before surgery, and intraoperative and postoperative variables will be prospectively collected for these patients. Preoperative anemia optimization protocols such as this have been assessed from a cost–benefit analysis perspective and have been found to be cost-saving on both an individual as well as a healthcare system level. Delaforce et al. demonstrated savings of about $13,000 per patient in their model [40]. Wan et al. similarly found a preoperative optimization protocol to be cost saving in a an orthopedic surgery population due to decreased length of stay and decreased transfusions [41].

## Conclusions

Preoperative anemia is strongly linked to an increased length of stay and utilization of healthcare resources after ASD correction. We found that patients with anemia had increased overall hospital LOS, increased ICU LOS, lower likelihood of discharge to home, and greater likelihood of reoperation. In this cohort, a Hgb of < 13.5 was associated with overall LOS, while Hgb < 13 was associated with ICU LOS and higher rates of re-intervention. These findings highlight the importance of preoperative optimization in patients undergoing surgery for ASD, and underscore the need to better understand the underlying pathophysiology of anemia in surgical cohorts.

## Data Availability

The datasets generated during and/or analyzed during the current study are not publicly available but are available from the corresponding author on reasonable request.
